# Molecular Diagnostics for Soil-Transmitted Helminths

**DOI:** 10.4269/ajtmh.16-0266

**Published:** 2016-09-07

**Authors:** Elise M. O'Connell, Thomas B. Nutman

**Affiliations:** ^1^ Laboratory of Parasitic Diseases, National Institute of Allergy and Infectious Diseases, National Institutes of Health, Bethesda, Maryland

## Abstract

Historically, the diagnosis of soil-transmitted helminths (STHs) (e.g., *Strongyloides stercoralis*, *Trichuris trichiura*, *Ancylostoma duodenale*, *Necator americanus*, and *Ascaris lumbricoides*) has relied on often-insensitive microscopy techniques. Over the past several years, there has been an effort to use molecular diagnostics, particularly quantitative polymerase chain reaction (qPCR), to detect intestinal pathogens. While some platforms have been approved by regulatory bodies (e.g., Food and Drug Administration) to detect intestinal bacteria, viruses, and protozoa, there are no approved tests currently available for STH. Although studies comparing qPCR to microscopy methods for STH are imperfect, due in large part to a lack of a sufficient gold standard, they do show a significant increase in sensitivity and specificity of qPCR compared with microscopic techniques. These studies, as well as the advantages and disadvantages of using qPCR for STH diagnosis, are discussed. Guidelines for those designing future studies utilizing qPCR are proposed for optimizing results, as is the proposition for using standardized molecular diagnostics routinely for STH in clinical laboratories and for field-based studies when possible.

## Introduction

Soil-transmitted helminths (STHs) encompass a number of intestinal parasitic nematodes that are acquired either by larvae burrowing through intact skin (*Strongyloides stercoralis*, the hookworms *Ancylostoma duodenale* and related spp., and *Necator americanus*) or by the fecal oral route (*Ascaris lumbricoides* and *Trichuris trichiura*). As a group, STH are on the World Health Organization's (WHO's) list of 17 neglected tropical diseases^1^ because of the significant morbidity they cause and their propensity to be poverty promoting. Although WHO does not include *S. stercoralis* on its formal list of STH, we include it here because it is highly prevalent and can be a significant cause of morbidity and mortality^2^ in low-, middle- and high-income countries alike.

Infections with STHs are often clinically asymptomatic, but they can be associated with eosinophilia and/or prolonged gastrointestinal symptoms, which most often occur in returning travelers^3^–^5^ and immigrants.^6^–^8^ These infections are often underdiagnosed given the decreasing number of well-trained personnel with the competence in identifying eggs and/or larvae in traditional stool-based microscopic methods and the intermittent shedding of eggs and/or larvae by some of these parasites (e.g., *Strongyloides*^6^,^9^,^10^).

Over the past 20 years, there has been an increasing effort to use molecular diagnostics for STH in epidemiologic studies and for the diagnosis of individual patients in some high-income countries.^11^,^12^ With the recent Food and Drug Administration (FDA) approval of several molecular diagnostic tools for stool bacterial and a few protozoa pathogens,^13^,^14^ the use of twenty-first century technology for the detection of intestinal pathogens has finally begun. However, there is no such FDA-approved molecular platform for diagnosing gastrointestinal helminth infections. In this review, we summarize the work that has been done in this growing field with specific attention paid to the strengths and limitations of using molecular diagnostics (particularly quantitative polymerase chain reaction [qPCR] platforms) in detecting STH.

## An Inadequate Gold Standard

Part of the difficulty in determining the sensitivity and specificity of qPCR and other molecular-based diagnostics is the lack of a sufficient gold standard against which to compare these newer techniques, given the general insensitivity of stool-based microscopic methods commonly in use. For example, the Kato-Katz (KK) technique was initially developed to detect *Schistosoma* spp. eggs,^15^ but is currently the most commonly used technique in STH surveys.^16^ It is particularly problematic in accurately detecting hookworm infections as the stool must be prepared immediately,^17^ and the clarification step (i.e., glycerin) can make the eggs unrecognizable.^18^ Interestingly, it is partly due to the lack of sensitivity of KK in identifying the larvae of *S. stercoralis* that has informed the decision by the WHO to not include this pathogen as an STH.^16^

Some studies have attempted to deal with this (lack of sufficient gold standard) problem by considering true positives to be the sum of the positives found by microscopy and/or PCR.^19^–^22^ Others have used several different microscopic detection methods with or without the use of statistical modeling to project the true prevalence, sensitivity, and specificity for any given method.^23^ Regardless of the method used, with rare exception, studies comparing microscopy to molecular methods (primarily qPCR) to diagnose STH have found markedly increased sensitivities with molecular diagnostics (Table 1). Moreover, because many of the molecular diagnostic techniques are multiplexed^24^,^42^,^43^ or multi-parallel,^19^,^21^,^22^ studies have also shown an increased ability to detect multiple concurrent infections using qPCR when compared with microscopy.^19^,^21^,^43^–^45^

## Applications of Qpcr as an STH Diagnostic

The WHO has set a worldwide goal to eliminate childhood morbidity caused by STH by 2020. Dictating which communities receive anthelmintic drugs, how often they receive them, and when community treatment is stopped is based on population prevalence rates determined by a small community sampling.^16^ Therefore, the very sensitive nature of qPCR (even post-treatment where eggs are often no longer visible), the improved ability to detect multiple helminths in a given sample and the ability to reproducibly quantitate egg burden, would be a huge asset to control programs.

Research studies are already utilizing qPCR for mass screening of stool samples for STH in malaria^46^ and in vaccine studies (currently underway) as stool can be stored relatively easily (see about preservatives in the section Technical Considerations in Using qPCR as an STH diagnostic) and sent offsite for qPCR not only for STH but also for protozoa and other gastrointestinal pathogens.

One of the most common criticisms of qPCR is the high cost compared with traditional methods. In one price comparison, a multiplex platform was estimated to cost about the same in consumables as the cost of microscopy,^45^ and consumable cost for multi-parallel singleplex qPCR of one group was estimated to be almost half the cost of microscopy^19^ on a per test basis. Thus, depending on the technique used, cost may not be a prohibitive factor.

## Technical Considerations in Using Qpcr As an STH Diagnostic

Despite the sensitive, rapid, and quantitative nature of qPCR for the diagnosis of STH, there are important methodological considerations in test design and results interpretation. Stool contains bile acids and other substances that inhibit the PCR product amplification.^47^ Early on in the use of molecular diagnostics for fecal pathogens, there were significant sensitivity problems when DNA extraction techniques that were not specifically designed to remove these inhibitors^48^ were used. PCR inhibitors are largely removed without problem using 1) more recent in-house developed protocols,^27^,^49^ 2) tissue kits with additional inhibitor removal steps,^24^,^50^ and 3) stool and soil-engineered kits for DNA extraction.^19^,^21^,^43^,^50^–^53^

A more clinically validated study will also amplify an internal control for each specimen to ensure efficient PCR inhibitor removal and to eliminate the possibility of false negatives in the molecular diagnostic results.^21^,^24^,^43^,^49^,^50^,^52^

Sensitivity can also be decreased by formalin fixation of the stool prior to DNA extraction.^54^ While specific stool preservation methods have not been directly compared in helminth detection through qPCR, in studies assessing the impact of different preservative measures on protozoal DNA amplification,^55^–^57^ it has been shown that samples stored in potassium dichromate can be stored at room temperature for prolonged periods before qPCR without any significant loss in sensitivity.

Unlike bacteria, which have cell walls that are easy to lyse, some physical stress is required to optimize the release of the nucleic acids from helminth eggs or larvae (and even some protozoa^58^). While freeze-thaw cycles, heating, and/or sonication do appear to offer an advantage over standard lysis buffer methods,^49^,^59^ the use of a tissue homogenization (“bead beating”) step with beads resistant to degradation (i.e., not glass) likely offers the most thorough disruption of parasite ova, thereby increasing molecular-based assay sensitivities significantly.^60^ The amount of stool extracted, the type of physical disruption method used, as well as the relative efficiency of commercial kits in extracting DNA^50^,^60^ likely explain much of the variability in sensitivity in published studies (see Table 1).

When interpreting molecular diagnostic results, it is also important to know the DNA sequence being amplified (see Table 2) and the limitations inherent in testing for a widely conserved sequence compared with sequence(s) that are species specific. In terms of qPCR platforms, singleplex (often using a multi-parallel approach^19^,^21^,^22^,^52^,^63^) offers slightly more sensitivity compared with multiplexed assays^24^,^42^,^44^,^53^,^64^,^65^(in which reagents in a given tube/well may be limiting). However, it may be possible to optimize a multiplex format to be equally sensitive to a singleplex approach.^43^ Multiplexed assays do, however, require more sophisticated and expensive equipment and labeled probes that may be less universally available (and more costly).

## Qpcr in the Detection of Each of the Major Sth

### Ascaris lumbricoides.

At an estimated prevalence rate of 819 million infections worldwide, *A. lumbricoides* is by far the most common STH.^66^ Several large studies have shown a clear relationship between stool DNA concentration as determined by qPCR and egg counts.^19^,^21^,^43^ Interestingly, there is also a good correlation between DNA quantification in the stool (presumably reflecting egg DNA) and the number of expelled adult worms after albendazole treatment.^21^ The evaluation of the existing data on *A. lumbricoides* across multiple studies (Table 1) has been aided enormously by the fact that all of the studies have targeted the internal transcribed spacer 1 region (see Table 2). In addition, of all the STH, KK identifies *A. lumbricoides* the most easily. More reliable reproducibility likely explains the relatively small gap (still in favor of the molecular diagnostics) in sensitivities between KK and qPCR for *Ascaris* (Table 1).

### Hookworms.

*Necator americanus* and *A. duodenale* are classically the two species of hookworm considered to be the most prevalent and clinically relevant worldwide. However, in certain regions of the world, *Ancylostoma ceylanicum*^30^ and *Oesophagostomum bifurcum*^24^ are very prevalent intestinal parasites, both of which are indistinguishable from *N. americanus* and *A. duodenale* using standard microscopic diagnostic methods. Thus, in areas where molecular testing has yet to characterize the exact species of infecting hookworms, the presumed predominant hookworm species may not always be present. This inability to accurately distinguish among the hookworms species morphologically has led to some confusion in that highly species-specific primer/probe combinations have led to some “false negatives” when comparing stool microscopy to qPCR.^17^,^61^ Indeed, egg-spiking experiments have determined that qPCR can detect a single hookworm egg in 200 mg of stool^36^ suggesting qPCR is extraordinarily sensitive, but the eggs seen by microscopy may not be the species that the primer/probe set was designed to detect.

Under optimal microscopic conditions, stool egg counts, stool larval counts, and clinical outcomes have been highly correlated with qPCR cycle times.^21^,^23^,^24^,^43^ For example, a study on hookworm infection in Malawian children found a significant interrelationship between the burden of hookworms as determined by qPCR, particularly *A. duodenale*, and the severity of iron deficiency and anemia.^67^

### Trichuris trichiura.

*Trichuris* infections in some populations are associated with iron deficiency anemia.^68^–^70^ The ova of *Trichuris* spp. are notorious for being more difficult to break open in the DNA extraction process than any other STH. Indeed, it is clear that a tissue homogenization step is imperative to achieve egg disruption.^71^ This parasite is the least well studied in the context of molecularly based diagnosis. However in the few studies where *T. trichiura* infection has been assessed,^19^,^22^,^52^ homogenization with or without a heating step has shown to yield highly sensitive qPCR results (Table 1), in one case predicting the limit of detection to be a single egg.^22^ Egg counts have also been shown to correlate highly when comparing microscopy to qPCR.^19^ Similar to hookworm, a number of *Trichuris* species besides *T. trichiura* are increasingly being recognized as having the potential to be a human pathogen.^72^ In cases where sequences highly specific for *T. trichiura* are targeted in qPCR, other related species (e.g., *Trichuris ovis*) may be missed.^22^

### Strongyloides stercoralis.

Strongyloidiasis, caused by *S. stercoralis*, is problematic due to the prolonged period maintained by this infection, as well as the potential for accelerated autoinfection with immune suppression (most often associated with corticosteroid use or human T-lymphotropic virus type 1 infection). Of all the intestinal helminths, it is certainly the one most difficult to diagnose through stool microscopy. This is due to the intermittent nature of larval shedding in the stool, as well as the relatively low numbers of larvae found in the stool (with the exception during accelerated autoinfection).^9^

Of the DNA sequences targeted (see Table 2), amplification of the 18S small subunit (Genbank accession number AY029262) has been proven to be more sensitive than cytochrome *c* oxidase 1 or the dispersed repeat sequence,^26^ and has been one of the most (if not the most) used sequence in qPCR for *S. stercoralis*.

One of the easiest and most sensitive methods for detecting infection with *S. stercoralis* is through the Koga agar plate culture, and so is often the method to which qPCR is compared. However, this test also can give a positive result in the setting of hookworm infection and can contribute to perceived false negatives and an overall low sensitivity seen in some studies using qPCR.^26^,^61^,^62^ However, in many epidemiologic surveys, qPCR has typically increased overall diagnostic yield by 2- to 8-fold when compared with a variety of microscopic techniques.^12^,^19^,^27^,^45^,^51^,^63^,^73^ Unlike the other STH, however, the quantification of larvae in the stool has not been shown to predict the adult worm burden, and so qPCR would not be used as a reflection of adult worm burden in a clinical setting.

## Proposed Guidelines in Using Qpcr to Diagnose STH in Research Studies

Given the variability in sensitivity and specificity of qPCR found in previous studies, we propose some guidelines for use in designing trials comparing qPCR with microscopy results. First, DNA extraction should be performed using a commercial stool or soil DNA extraction kit, or published protocols validated for use in stool-based PCR. A physical disruption step early in DNA extraction (use of a tissue homogenizer with ceramic or zirconia/silica beads, particularly if attempting to detect *Trichuris* spp.) should be used. To exclude the possibility of false-negative results due to PCR inhibitors, samples should be spiked with an internal control and amplified along with target sequences of interest. Only validated primer/probe sequences should be used for a particular target. Alternatively, one can validate new sequences through Basic Local Alignment Search Tool^74^ searches. Once identified, the primers/probes derived from these new targets can be tested using genomic DNA from the species of interest and from potentially cross-reactive organisms to determine specificity. In performing stool PCR surveys, one should ideally know the endemic hookworm and *Trichuris* species in the population to be tested based on previous molecular testing/sequencing; if unavailable it might be important to include a pilot discovery phase to determine the actual species of infecting parasitic helminth found in the stool.

## Conclusion

The highly sensitive, rapid, and scalable nature of qPCR makes its utilization in diagnosing STH extremely appealing over insensitive and labor-intensive traditional microscopic methods. We have suggested here its superiority over microscopy methods (particularly KK) in sensitivity while still providing a quantitative measure of infection intensity. There are, however, important technical lessons that have been learned that provide a framework to maximize the utility of this potentially valuable molecular approach, which we have highlighted here. Until now molecular detection of STH has been restricted to the research setting. Given the neglected nature of STH and the large cost of the regulatory processes, moving forward in developing a FDA- or European Union-approved platform would require significant political will and/or philanthropic efforts. Nevertheless, given that surveillance and monitoring for many other pathogens are being done using standardized (but not commercialized) molecular techniques, we would argue that use of qPCR in detecting STH would likely provide a more accurate and cost-effective approach to the WHO STH elimination strategy and should be considered seriously.

## Figures and Tables

**Table 1:** Targets and unique primer sequences reported in the literature for PCR for STH*

Organism	Target region	Citations of unique primer sequences
*Strongyloides stercoralis*	ITS-1	25
	18S	26
	Cytochrome oxidase 1	26,27
	Sequence repeats	22,26
	ITS-1, 5.8S, ITS-2	28
*Ancylostoma duodenale/ceylanicum*	ITS-2	24,29
	ITS-1, 5.8S, ITS-2	30,31
*A. duodenale*	Cytochrome oxidase 1	32
	Sequence repeats	22
*Ascaris lumbricoides*	ITS-1	22,33,34
	Cytochrome oxidase 1	35
*Necator americanus*	ITS-2	24,36–39
	ITS-1, 5.8S, ITS-2	30,31,39,40
	Cytochrome oxidase 1	32
	Sequence repeats	22
*Trichuris trichiura*	ITS-1	19
	ITS-2	41
	Sequence repeats	22

ITS = internal transcribed spacer; qPCR = quantitative polymerase chain reaction; STH = soil-transmitted helminth.

* Included are studies utilizing conventional, nested, qPCR.

**Table 2:** Reported sensitivities of qPCR for diagnosing STH compared with reported stool microscopy sensitivities*

Organism	Sensitivities reported by qPCR	Sensitivities by microscopy
*Ascaris lumbricoides*	85.7%,†^20^ 98%,**^21^ 100%^52^	71.4%,†^20^ 70,** 21 88%^52^
Hookworms	96.9%,†^20^ 78.9%,^61^ 75.7–83.3%,¶∥^23^ 98%,^21^ 100%^36^	31.3%,†^20^ 79.2–88.8%,∥^23^ 32%**^21^
*Strongyloides stercoralis*	76%,¶^62^ 83.3%,†^20^ 83.3–88.9%,§^61^ 11.6%,¶∥^23^ 93.8%,‡‡^51^ 86.4%,§∥^26^ 84.7%††^27^	16.7%,†^20^ 50%,‡^62^ 28.3%∥^23^
*Trichuris trichiura*	100%^52^	88%^52^

qPCR = quantitative polymerase chain reaction; STH = soil-transmitted helminth.

* Unless otherwise stated, gold standard in determining true positives was the sum of positives by qPCR and microscopy method used. Study specific microscopy methods (if described) are listed below.

† Single stool sample prepared with a combination of Kato-Katz, wet preparation, and formol-ether concentration methods.

‡ Single stool sample subjected to Baermann funnel concentration and Koga agar plate culture.

§ Sensitivity excludes those positive by Koga agar and negative by Baermann funnel. See text for details.

¶ No internal controls used to rule out PCR inhibition in this study.

∥ Mathematical modeling to determine gold standard taking into account results of single stool subjected to FLOTAC, Kato-Katz, Baermann, and qPCR.

** Single stool sample prepared by Kato-Katz, with duplicate slides assessed by two technicians.

†† Gold standard a combination of nested PCR, Koga agar plate culture, and formalin ether concentration.

‡‡ Single stool sample subjected to formalin-ethyl acetate concentration, agar plate culture, and Harada Mori technique.
